# Impact of empagliflozin on right ventricular parameters and function among patients with type 2 diabetes

**DOI:** 10.1186/s12933-021-01390-8

**Published:** 2021-10-04

**Authors:** Bradley Sarak, Subodh Verma, C. David Mazer, Hwee Teoh, Adrian Quan, Richard E. Gilbert, Shaun G. Goodman, Karan Bami, Otávio R. Coelho-Filho, Vineeta Ahooja, Djeven P. Deva, Vinay Garg, Sumeet Gandhi, Kim A. Connelly, Andrew T. Yan

**Affiliations:** 1 Division of Cardiology, Terrence Donnelly Heart Centre, St Michael’s Hospital, 30 Bond Street, Toronto, ON M5B 1W8 Canada; 2 University of Toronto, Toronto, Canada; 3 Keenan Research Centre, Li Ka Shing Knowledge Institute, St Michael’s Hospital, Toronto, Canada; 4 Division of Cardiac Surgery, St Michael’s Hospital, Toronto, Canada; 5 Department of Anesthesia, St Michael’s Hospital, Toronto, Canada; 6 Division of Endocrinology and Metabolism, St Michael’s Hospital, Toronto, Canada; 7 Department of Internal Medicine, Discipline of Cardiology, State University of Campinas, Campinas, Brazil; 8 Heart Health Institute, Toronto, ON Canada; 9 Department of Medical Imaging, St. Michael’s Hospital, Toronto, Canada; 10 Trillium Health Partners, Toronto, Canada

**Keywords:** Type 2 diabetes, Right ventricle, Sodium-glucose transporter 2 inhibition

## Abstract

**Background:**

Sodium-glucose cotransporter 2 (SGLT2) inhibition reduces cardiovascular events in type 2 diabetes (T2DM) and is associated with a reduction in left ventricular (LV) mass index. However, the impact on right ventricular (RV) remodeling is unknown. Accordingly, the objective of this study was to assess the impact of SGLT2 inhibition on RV parameters and function in T2DM and coronary artery disease (CAD).

**Methods:**

In EMPA-HEART CardioLink-6, 97 patients with T2DM and CAD were randomly assigned to empagliflozin 10 mg (n = 49) once daily or placebo (n = 48). Cardiac magnetic resonance imaging was performed at baseline and after 6 months. RV mass index (RVMi), RV end-diastolic and end-systolic volume index (RVEDVi, RVESVi) and RV ejection fraction (RVEF) were assessed in blinded fashion.

**Results:**

At baseline, mean RVMi (± SD) (11.8 ± 2.4 g/m^2^), RVEF (53.5 ± 4.8%), RVEDVi (64.3 ± 13.2 mL/m^2^) and RVESVi (29.9 ± 6.9 mL/m^2^) were within normal limits and were similar between the empagliflozin and placebo groups. Over 6 months, there were no significant differences in RVMi (− 0.11 g/m^2^, [95% CI − 0.81 to 0.60], p = 0.76), RVEF (0.54%, [95% CI − 1.4 to 2.4], p = 0.58), RVEDVi (− 1.2 mL/m^2^, [95% CI − 4.1 to 1.7], p = 0.41) and RVESVi (− 0.81 mL/m^2^, [95% CI − 2.5 to 0.90], p = 0.35) in the empaglifozin group as compared with the placebo group. In both groups, there was no significant correlation between RVMi and LVMi changes from baseline to 6 months.

**Conclusions:**

In this post-hoc analysis, SGLT2 inhibition with empagliflozin had no impact on RVMi and RV volumes in patients with T2DM and CAD. The potentially differential effect of empagliflozin on the LV and RV warrants further investigation.

*Clinical Trial Registration*: URL: https://www.clinicaltrials.gov/ct2/show/NCT02998970?cond=NCT02998970&draw=2&rank=1. Unique identifier: NCT02998970.

## Introduction

The sodium-glucose cotransporter 2 (SGLT2) inhibitor empagliflozin reduces cardiovascular (CV) mortality, all-cause mortality and heart failure (HF) hospitalization in patients with type 2 diabetes (T2DM) and established atherosclerotic CV disease (ASCVD) [1]. Other SGLT2 inhibitors have also been evaluated in large CV outcome trials in those at risk for, or with established ASCVD, and have shown similar results [2–5]. More recently, the benefit of empagliflozin and dapagliflozin was confirmed in patients with HF with reduced ejection fraction with or without T2DM [6–8].

The mechanism of these benefits, particularly on reducing HF hospitalizations and CV death, remains unclear. In diabetic kidney disease, canagliflozin was associated with attenuated or decreased levels of biomarkers that suggest an effect on molecular processes related to inflammation, the extracellular matrix and fibrosis [9]. Other proposed non-atherothrombotic mechanisms of SGLT2 inhibition include natriuresis, osmotic diuresis, a reduction in preload and afterload, and inhibition of the cardiac sodium-hydrogen exchanger [10–12]. However, whether and how these mediators alter cardiac structure and function remain incompletely understood.

The EMPA-HEART CardioLink-6 trial demonstrated that compared with placebo, the addition of empagliflozin to antihyperglycemic treatment in individuals with T2DM and coronary artery disease was associated with a significant reduction in left ventricular (LV) mass index (LVMi) as measured by cardiac magnetic resonance imaging (cMRI). Treatment with empagliflozin was also associated with a significant lowering of ambulatory systolic blood pressure with no impact on the circulating levels of NT-pro B-type natriuretic peptide (NT-pro-BNP) [13].

cMRI provides the reference standard assessment of right ventricular (RV) structure and function [14], which are prognostic markers in various clinical settings including ischemic cardiomyopathy [15], non-ischemic cardiomyopathy [16], HF [17] and a multiethnic population free of CV disease [18]. T2DM affects RV remodeling, systolic and diastolic function, even in the setting of preserved LV ejection fraction (LVEF) [19–21]. Furthermore, T2DM is associated with RV dysfunction following ST elevation myocardial infarction [22]. However, the effects of SGLT2 inhibition on RV structure and function are unknown.

The primary objective of this post-hoc analysis of the EMPA-HEART CardioLink-6 was to assess using cMRI whether empagliflozin alters RV parameters, function and remodeling among patients with T2DM and established coronary artery disease. The secondary objective was to examine the relationships of RV remodeling with LV remodeling, blood pressure and select cardiac biomarkers.

## Methods

### Trial design

This study is a post-hoc analysis of the EMPA-HEART (Effects of Empagliflozin on Cardiac Structure in Patients with Type 2 Diabetes) CardioLink-6 trial (Unique identifier: NCT02998970), the details of which have been described [13]. In short, EMPA-HEART CardioLink-6 was a single centre, double-blind, randomized, placebo-controlled, investigator-initiated phase IV trial of empagliflozin in 97 adult patients with T2DM, HbA1c ≥ 6.5% and ≤ 10% on stable background antihyperglycemic therapy, estimated glomerular filtration rate ≥ 60 mL/min/1.73^2^ and previous myocardial infarction or coronary revascularization. At baseline, participants underwent clinical and laboratory assessment, and were evaluated with 24-h ambulatory blood pressure monitoring as well as cMRI. Recruited patients were randomized in a 1:1 ratio to 6 months of empagliflozin 10 mg daily or placebo in addition to standard of care. Clinical visits were performed three times over the 6-month follow-up period. Standard transthoracic echocardiography was performed at baseline and 6 months. Right ventricular systolic pressure (RVSP), tricuspid regurgitation peak velocity, tricuspid annular plane systolic excursion (TAPSE) and RV S’ were assessed. The final visit included a repeat cMRI and 24-h ambulatory blood pressure monitoring.

### Cardiac magnetic resonance imaging

cMRI scans were performed using a standardized protocol at baseline and 6 months using a clinical 3T MRI scanner (MAGNETOM Skyra; Siemens Healthcare, Erlangen, Germany). A steady-state free-precession sequence was used for standard cine imaging covering the entire LV and RV, typically with 8–12 contiguous short-axis images without interslice gap. Images were acquired in the supine position and at end–expiration.

**Fig. 1:**
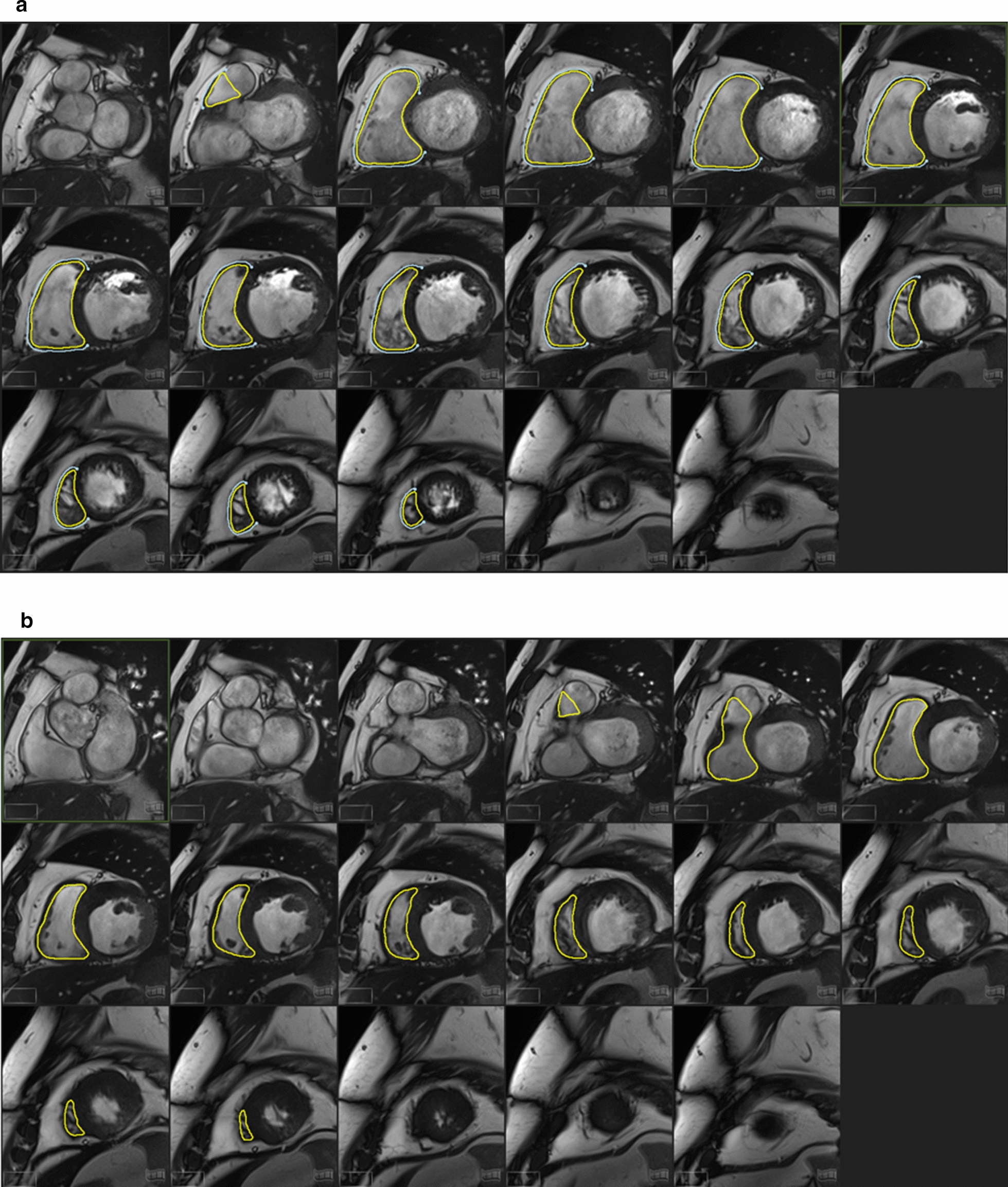
Example of contouring of the right ventricular endocardium (yellow) and epicardium (blue) in one patient at end-diastole (**a**) and end-systole (**b**). Endocardial borders at end–diastole and at end–systole in contiguous short–axis images were manually traced. The epicardial borders at end–diastole were also traced. The difference in area at end-diastole was multiplied by the slice thickness and the sum of these differences throughout the entire right ventricle was multiplied by the myocardial specific density to calculate right ventricular mass. Right ventricular trabeculations were considered part of the blood pool

Image post-processing was performed offline using CVi 42 (Circle Cardiovascular, Calgary, Alberta, Canada) and was blinded to clinical data as well as the timing of image acquisition. Contouring was performed by a single cardiovascular imaging fellow (BS), over read by two level 3 cMRI readers (ATY and KAC). Endocardial borders at end–diastole and at end–systole in contiguous short-axis images were manually traced (Fig. 1). The epicardial borders at end–diastole were also traced. The difference in area at end-diastole was multiplied by the slice thickness and the sum of these differences throughout the entire RV was multiplied by the myocardial specific density (1.05 g/cm^3^) to calculate the RV mass (RVM). RV trabeculations were considered part of the blood pool and not as a part of the RVM. RV end-diastolic and end-systolic volumes (RVEDV and RVESV) were determined by summing the volume across slices without geometric assumptions, and RV ejection fraction (RVEF) was calculated as (RVEDV-RVESV)/RVEDV × 100%).

### Study outcomes

In this substudy, the primary outcome measure was the change in RVM from baseline to 6 months indexed to the body surface area at baseline (RVMi). Secondary outcomes included the baseline to 6-month changes in RVEDV and RVESV indexed to body surface area (RVEDVi, RVESVi), and RV ejection fraction (RVEF).

### Statistical analysis

All analyses followed an intent-to-treat approach. Categorical variables were presented using counts and percentages and were compared using the χ^2^ test. Continuous variables are presented as either mean (standard deviation) and compared with the Student’s t-test, or as median (interquartile range) and compared with the Kruskal–Wallis test. Spearman’s (nonparametric) correlation was used to assess the relationships between RV parameters and LV parameters, blood pressure, NT-pro-BNP and high sensitivity troponin I. Analysis of covariance (ANCOVA) was used to compare primary and secondary outcomes between the two randomized arms, adjusting for the baseline measurements. Intra-observer variability in the measurement of RV parameters was assessed by intraclass correlation coefficient for absolute agreement using 20 randomly selected studies. Analysis was performed using SPSS 25 (IBM) and statistical significance was set at a two-sided p value < 0.05.

## Results

Of the 97 participants enrolled, 49 were assigned to empagliflozin 10 mg/day and 48 to placebo. Among those randomized, 6-month outcome data were unavailable for 7 (5 in the treatment group and 2 in the placebo group). Of these, 3 patients refused and 2 did not undergo follow-up cMRI. Two other patients were lost to follow-up. Accordingly, both baseline and 6-month cMRI were available for 44 participants in the empagliflozin group and 46 in the placebo arm.

The demographics and baseline characteristics of those randomized were previously published [13] and are summarized in Table 1. Mean (SD) baseline LVMi and LVEF were 59.3 (10.9)g/m^2^ and 58.0 (7.5)% in the empagliflozin group, and 62.2 (12.8)g/m^2^ and 55.5 (8.7)% in the placebo group, respectively. The change in LVMi from baseline to 6 months was − 2.6 (7.8)g/m^2^ for the empagliflozin group and − 0.01 (5.7)g/m^2^ for the placebo group (adjusted between group difference − 3.35 g/m^2^, 95% CI [− 5.9, − 0.81], p = 0.01). There was no significant change in LV end-systolic or end-diastolic indices or LVEF from baseline to 6 months.

**Table 1:** Baseline characteristics of the study population

	Empagliflozin 10 mg (n = 44)	Placebo (n = 46)
Age, years*	64 (57, 69)	64 (56, 72)
Body mass index, kg/m^2^*	26.7 (24.5, 30.2)	26.6 (24.4, 29.3)
Body surface area, m^2^*	2.0 (1.8, 2.1)	1.9 (1.8, 2.1)
Male sex	90	96
Smoking history	41	46
Duration of type 2 diabetes, years*	10.0 (4.0, 15.0)	10.0 (5.0, 15.0)
Hypertension	92	90
History of myocardial infarction	39	44
History of percutaneous coronary intervention > 2 months before screening	53	40
History of coronary artery bypass surgery > 2 months before screening	57	56
History of heart failure	4.0	8.0
History of peripheral artery disease	4.0	6.0
History of transient ischemic attack or stroke	16	13
Serum creatinine, mg/dL*	0.9 (0.8, 1.0)	0.9 (0.8, 1.0)
Hemoglobin A1c, %*	7.9 (7.5, 8.4)	7.9 (7.3, 8.7)
Hematocrit, %*	0.42 (0.40, 0.46)	0.42 (0.39, 0.44)
Systolic blood pressure, mmHg*	128 (120, 143)	134 (125, 146)
Diastolic blood pressure, mmHg*	74 (69, 82)	77 (71, 81)
Heart rate, bpm*	67 (60, 77)	68 (60, 76)
Cardiac MRI data		
Left ventricular mass, g	116.5 (26.3)	120.9 (33.0)
Left ventricular mass index, g/m^2^	59.3 (10.9)	62.2 (12.8)
Left ventricular end diastolic volume index, mL/m^2^	63.3 (15.5)	71.4 (15.4)
Left ventricular end systolic volume index. mL/m^2^	27.1 (10.5)	32.3 (11.8)
Left ventricular ejection fraction. %	58.0 (7.5)	55.5 (8.7)
Echocardiographic data		
Right ventricular systolic pressure, mmHg†	22.8 (6.5)	20.7 (4.4)
Peak tricuspid regurgitation velocity, m/s†	2.2 (0.38)	2.1 (0.27)
Tricuspid annular plane systolic excursion, cm	2.0 (1.2)	1.8 (0.5)
Right ventricular S’, mm	10.4 (2.7)	10.9 (2.9)
Biomarkers		
NT-pro B-type natriuretic peptide, pg/mL*	97.0 (46.0, 190)	116 (59.0, 230)
High sensitivity troponin I, ng/mL*	0.03 (0.03, 0.20)	0.03 (0.03, 0.03)
Medications at baseline		
Aspirin/P2Y12 inhibitor	82	85
Beta blocker	78	81
Calcium channel blocker	12	31
Angiotensin converting enzyme inhibitor/angiotensin receptor blocker	82	85
Statin	96	96
Insulin	25	25
Metformin	96	92

Data expressed as percentages or mean (standard deviation) unless otherwise specified

*Median (25th, 75th percentile)

†Data available for 14 in the empagliflozin group and 17 in the placebo group

**Fig. 2:**
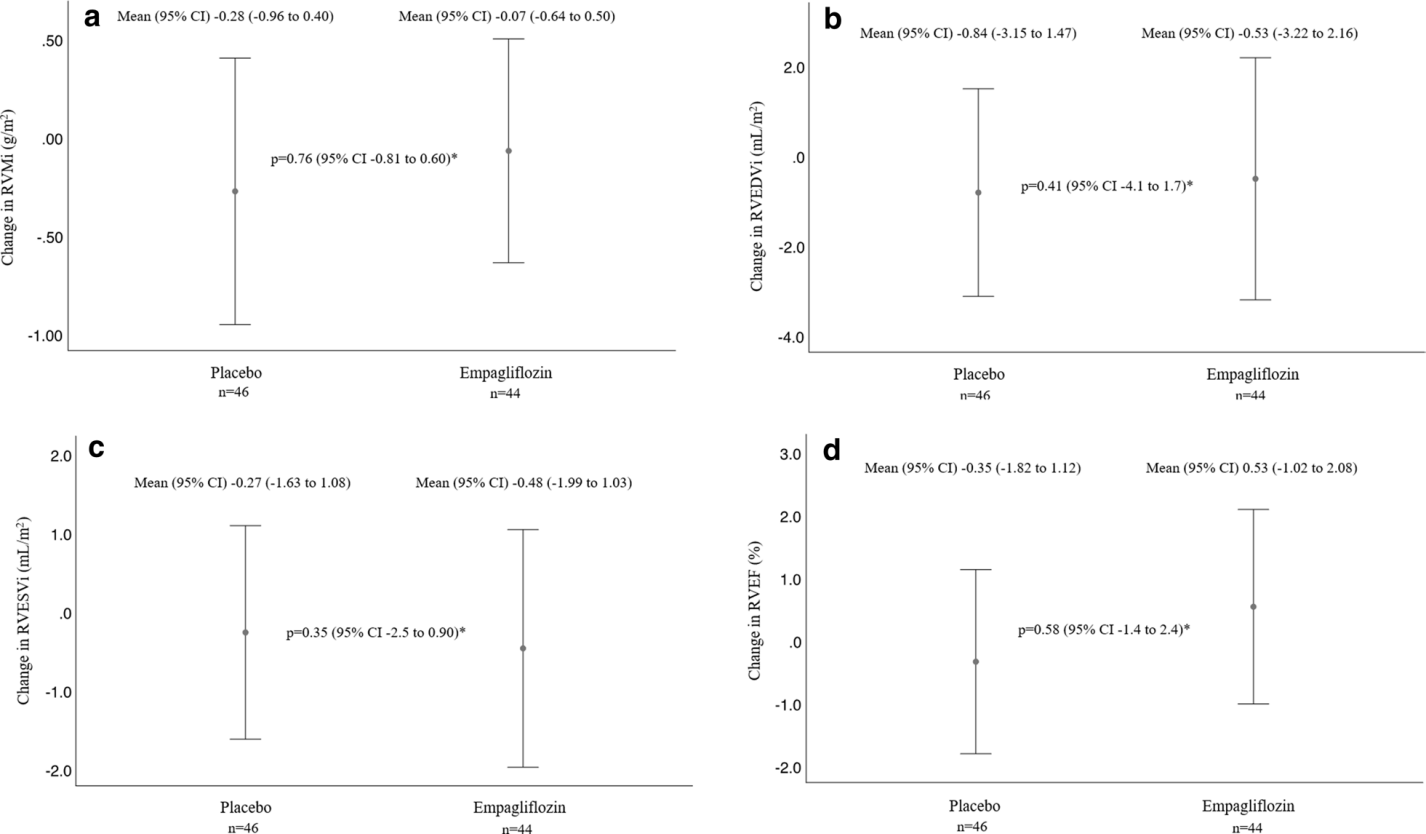
**a** Six-month mean changes in RVMi following treatment with empagliflozin versus placebo. **b** Six-month mean changes in RVEDVi following treatment with empagliflozin versus placebo. **c** Six-month mean changes in RVESVi following treatment with empagliflozin versus placebo. **d** Six-month mean changes in RVEF following treatment with empagliflozin versus placebo. (*) Data analyzed using ANCOVA adjusting for baseline values. RVMi, right ventricular mass index; RVEDVi, right ventricular end diastolic volume index; RVESVi, right ventricular end systolic volume index; RVEF, right ventricular ejection fraction. Baseline and 6-month data were available for 44 individuals in the in the empagliflozin group and 46 in the placebo group. Mean changes in RVMi, RVEDVi, RVESVi and RVEF are presented as mean (95% CI), and the adjusted differences between groups are shown with 95% CI. Data were analyzed using ANCOVA adjusting for baseline values

Mean (SD) baseline RVMi was 11.5 (2.35)g/m^2^ and 12.8 (2.37)g/m^2^ in the empagliflozin and placebo groups, respectively (Table 2). Mean RVMi, RVEF, RV diastolic and systolic volumes were all within normal limits [23]. Over 6 months, there were no significant differences in RVMi (− 0.11 g/m^2^, [95% CI − 0.81 to 0.60], p = 0.76), RVEF (0.54%, [95% CI − 1.4 to 2.4], p = 0.58), RVEDVi (− 1.2 mL/m^2^, [95% CI − 4.1 to 1.7], p = 0.41) and RVESVi (− 0.81 mL/m^2^, [95% CI − 2.5 to 0.90], p = 0.35) in the empaglifozin group as compared with the placebo group by ANCOVA adjusting for baseline values. Intraclass correlation coefficient values for absolute agreement in the measurement of RV parameters were > 0.95 for RVMi, RVEDVi, RVESVi and 0.86 for RVEF. Adjusted between group differences (mean, 95% CI) using ANCOVA are displayed in Table 2 and Fig. 2a–d.

**Table 2:** Changes in cMRI measured RV parameters following treatment for 6 months with either empagliflozin or placebo

		Empagliflozin 10 mg (n = 44)*			Placebo (n = 46)*		
		Baseline	6 months	Mean change (95% CI)	Baseline	6 months	Mean change (95% CI)
RV mass index, g/m^2^		11.5 (2.35)	11.4 (2.13)	− 0.07 (− 0.64 to 0.50)	12.8 (2.37)	11.8 (1.84)	− 0.28 (− 0.96 to 0.40)
RV end diastolic volume index, mL/m^2^		62.0 (13.2)	61.7 (10.8)	− 0.53 (− 3.22 to 2.16)	66.4 (12.6)	65.5 (10.4)	− 0.84 (− 3.15 to 1.47)
RV end systolic volume index, mL/m^2^		28.9 (6.51)	28.6 (6.24)	− 0.48 (− 1.99 to 1.03)	30.8 (6.98)	30.4 (5.60)	− 0.27 (− 1.63 to 1.08)
RV ejection fraction, %		53.2 (4.93)	53.7 (5.17)	0.53 (− 1.02 to 2.08)	53.8 (4.59)	53.5 (5.13)	− 0.35 (− 1.82 to 1.12)

*RV* right ventricle

Data expressed as percentages or mean (standard deviation) unless otherwise specified

*All p-values are non-significant

Mean (SD) baseline RVSP and tricuspid regurgitation peak velocity were 22.8 (6.5) mmHg and 2.2 (0.38) m/s in 14 patients in the empagliflozin group, and 20.7 (4.4) mmHg and 2.1 (0.27) m/s in 17 patients in the placebo group, respectively. The remaining subjects had missing data due to an insufficient tricuspid regurgitation spectral Doppler profile. Echocardiographic parameters that assess RV function were normal at baseline and unchanged at 6 months: − 0.099 (95% CI [− 2.8 to 0.08], p = 0.28) for TAPSE and − 0.32 (95% CI [− 1.2 to 0.52], p = 0.45) for RV S’.

The relationships between RV parameters and LV parameters, blood pressure and biomarkers were evaluated. In both groups, there was no significant correlation between RVMi and LVMi changes from baseline to 6-month follow up (Table 3). However, there was a significant correlation between the changes in RV and LV end-systolic and end-diastolic indexed volumes. The change in RVEF and LVEF were correlated in the control group but not the empagliflozin group. There was no significant correlation between changes in RV parameters with changes in NT-pro-BNP, high sensitivity troponin I, systolic or diastolic blood pressure (Table 4).

**Table 3:** Spearman’s correlation coefficients for the relationships between changes in right and left ventricular indices over 6 months

	Both groups (n = 90)		Empagliflozin 10 mg (n = 44)		Placebo (n = 46)	
	Spearman’s correlation	p-value	Spearman’s correlation	p-value	Spearman’s correlation	p-value
Mass index, g/m^2^	0.16	0.14	0.30	0.05	0.07	0.64
End diastolic volume index, mL/m^2^	0.58	< 0.001	0.52	< 0.001	0.54	< 0.001
End systolic volume index, mL/m^2^	0.43	< 0.001	0.36	0.02	0.44	0.002
Ejection fraction, %	0.30	0.01	0.13	0.40	0.43	0.003

**Table 4:** Spearman’s correlation coefficients between 6-month changes in right ventricular indices, biomarkers and blood pressure

Biomarker	Empagliflozin 10 mg (n = 44)*				Placebo (n = 46)*			
	RVEDVI	RVESVI	RVMI	RVEF	RVEDVI	RVESVI	RVMI	RVEF
NT-pro-BNP, pg/mL	0.04	− 0.06	0.06	0.11	− 0.05	− 0.18	0.06	0.15
High sensitivity troponin I, ng/mL	0.19	0.26	0.17	− 0.16	− 0.02	− 0.15	0.07	0.26
Systolic blood pressure	0.03	0.06	0.04	0.03	0.15	0.26	− 0.02	− 0.21
Diastolic blood pressure	− 0.10	− 0.09	0.01	0.06	0.06	0.13	0.12	− 0.19

NT-pro-BNP, NT-pro B-natriuretic peptide

*All p-values are non-significant

## Discussion

In this post-hoc analysis of the EMPA-HEART CardioLink-6 trial, the addition of empagliflozin to stable antihyperglycemic therapy in patients with T2DM and established coronary artery disease without HF did not result in significant changes in RVMi, RV volumes or RVEF over 6 months, as measured by cMRI. To our knowledge, this is the first cMRI study to assess changes in RV parameters and function in a randomized controlled trial of an SGLT2 inhibitor.

There has been considerable work conducted to delineate how SGLT2 inhibition affects the LV [24, 25]. The EMPA-HEART CardioLink-6 trial demonstrated that after 6 months of treatment with empagliflozin, there was a decrease in LVMi without a change in LV volumes or LVEF [13]. A substudy of this trial showed that empagliflozin exposure also resulted in a decrease in extracellular compartment volume as measured by cMRI [26]. In the SUGAR-DM-HF trial, which enrolled a more advanced HF population, empagliflozin also reduced LV end-diastolic and end-systolic volume index [27].

The RV is considerably different from the LV with respect to its structure, function, loading conditions and adaptation in states of disease [28]. Importantly, there is a paucity of information on how SGLT2 inhibition affects the morphology and function of the RV [29]. This is surprising given that individuals with T2DM may have impaired RV systolic and diastolic function as well as decreased RV volumes, even in the absence of established coronary artery disease or HF [19, 21, 30, 31]. Furthermore, T2DM is associated with RV systolic and diastolic dysfunction in the setting of HF with preserved LVEF, independent of RV afterload [20]. The underlying mechanism for this association remains unclear and has been postulated to be a consequence of hyperglycemia and the deposition of glycosylation products, as well as hyperinsulinemia resulting in myocyte hypertrophy, myocardial steatosis and inflammation [31].

The clinical relevance of RVMi in left-sided HF is not well established, and our current understanding is largely derived from other patient populations that included those with pulmonary hypertension [32]. In the current analysis, empagliflozin 10 mg daily for 6 months did not appear to have any effect on RVMi. This observation contrasts with the decrease in LVMi previously observed in the same study cohort [13]. While this difference could be attributed to the RV not being exposed to systemic afterload, it should be noted that the LVMi regression occurred independent of blood pressure changes. These discordant findings suggest that there may be other mechanisms, independent of afterload, which differentially affect the LV, RV, systemic and pulmonary vasculature.

In the Multi-Ethnic Study of Atherosclerosis, RVM by cMRI was positively associated with systolic blood pressure in 4204 individuals who were free of CV disease [33]. In a cMRI study that assessed RV remodeling in 25 hypertensive patients, systemic hypertension was also associated with greater RVMi and concentric RV remodeling. Although these changes were associated with LV remodeling, it was not reported if there was any correlation with pulmonary artery pressure [34]. Conversely, in our study, there was no significant correlation between changes in RVMi and changes in systemic systolic or diastolic blood pressure, despite a decrease in blood pressure in the group randomized to empagliflozin.

It remains unknown whether SGLT2 inhibition may have an effect on the RV in left-sided HF with combined pre- and post-capillary pulmonary hypertension. Animal models provide some insight with regard to pulmonary artery hypertension. In one study, empagliflozin significantly improved survival in rats with monocrotaline-induced pulmonary artery hypertension while reducing mean pulmonary artery pressure; this was accompanied by reduced RV hypertrophy and fibrosis [35]. The EMBRACE-HF trial showed that in patients with NYHA class III-IV HF and CardioMEMS pulmonary artery pressure sensors (mean pulmonary artery diastolic pressure 22 mmHg, median NT-pro-BNP 637 pg/mL), empagliflozin decreased pulmonary artery pressures independent of loop diuretic therapy [36]. RV remodeling in this context was not investigated, however, the change in mean and diastolic pulmonary artery pressure was under 2 mmHg, which would not be expected to meaningfully impact RV parameters and function.

Our cohort likely had a low prevalence of group 2 pulmonary hypertension since RVSP and tricuspid regurgitation peak velocity values were not elevated [37]. Moreover, NT-pro-BNP levels were within the normal range [38] and patients with HF were excluded. In the EMPA-HEART CardioLink-6 echocardiographic substudy, the majority of patients demonstrated only grade 1 diastolic dysfunction with mostly normal left atrial size [39], suggesting that our study population had normal filling pressures; our findings should not be extrapolated to those with pulmonary hypertension or HF. While SGLT2 inhibition may impact ventricular interdependence by altering filling pressures, diastolic function, systolic blood pressure and pulmonary pressures, the effect should not be significant in this cohort. However, it is likely to be more important in other populations and under different loading conditions.

RV volumes and RVEF in the EMPA-HEART cohort were in the normal range, as were LV volumes and LVEF. Moreover, NT-pro-BNP values were normal. Indeed, in the SUGAR-DM-HF trial, which enrolled patients with T2DM, LVEF < 40%, and NYHA class II-IV symptoms, empagliflozin reduced LV end-diastolic and end-systolic volume index as measured by cMRI [27]. RV parameters were not reported. It is plausible that the effect of SGLT2i on the RV, and even mechanism of action, may differ depending on the stage of HF. Our findings should not be extrapolated to those with pulmonary hypertension or advanced HF, particularly with RV dysfunction or hypertrophy, as this is beyond the scope of our study.

Our study has a number of limitations. First, the sample size was small and most of the participants were men, which may be relevant since the relationship between RV volumes and T2DM may differ between sexes [19]. Although the EMPA-HEART CardioLink-6 trial was not powered to assess for changes in the RV, the narrow 95% confidence intervals afforded by cMRI effectively ruled out clinically significant changes in RVMi despite the small sample size. Second, follow up duration was only 6 months. Third, there are technical challenges while contouring the RV including its thinner wall, delineation of the basal slice and artifacts including from sternotomy wires. Fourth, invasive pulmonary pressures were not known and an adequate tricuspid regurgitation spectral Doppler profile was only available in about one third of patients to estimate RVSP. Fifth, tissue mapping of the RV was not performed as part of this study.

There are also important strengths. First, to the best of our knowledge, this is the first substudy of a randomized placebo-controlled trial utilizing cMRI to assess the effect of SGLT2 inhibition on RV parameters and function. Second, cMRI is the gold standard assessment of the RV and is independent of geometric assumption. Image analysis was performed with blinding and good intra-observer variability, and all RV measurements were made independent of LV measurements.

## Conclusion

In this post-hoc analysis of the EMPA-HEART CardioLink-6 trial, in contrast to the LV, SGLT2 inhibition with empagliflozin had no impact on RVMi in patients with T2DM, coronary artery disease and normal LVEF. The potentially differential effect of empagliflozin on the LV and RV warrants further investigation.

## Data Availability

All data generated or analysed during this study are included in this published article (an its Additional files).

## Abbreviations

ANCOVA: Analysis of covariance
ASCVD: Atherosclerotic cardiovascular disease
CV: Cardiovascular
cMRI: Cardiac magnetic resonance imaging
EDV(i): End systolic volume (index)
EF: Ejection fraction
ESV(i): End systolic volume (index)
HF: Heart failure
LV: Left ventricle
LVM(i): Left ventricular mass (index)
NT-pro-BNP: NT-pro B-type natriuretic peptide
RV: Right ventricle
RVM(i): Right ventricular mass (index)
RVSP: Right ventricular systolic pressure
SGLT2: Sodium-glucose transporter 2
TAPSE: Tricuspid annular plane systolic excursion
T2DM: Type 2 diabetes
